# The effect of sampling density and study area size on landscape genetics inferences for the Mississippi slimy salamander (*Plethodon mississippi*)

**DOI:** 10.1002/ece3.7481

**Published:** 2021-05-01

**Authors:** Stephanie M. Burgess, Ryan C. Garrick

**Affiliations:** ^1^ Department of Biology University of Mississippi Oxford MS USA

**Keywords:** gene flow, herpetofauna, landscape genetics, microsatellites, sampling density, sampling effort, study area size

## Abstract

In landscape genetics, it is largely unknown how choices regarding sampling density and study area size impact inferences upon which habitat features impede vs. facilitate gene flow. While it is recommended that sampling locations be spaced no further apart than the average individual's dispersal distance, for low‐mobility species, this could lead to a challenging number of sampling locations, or an unrepresentative study area. We assessed the effects of sampling density and study area size on landscape genetic inferences for a dispersal‐limited amphibian, *Plethodon mississippi*, via analysis of nested datasets. Microsatellite‐based genetic distances among individuals were divided into three datasets representing sparse sampling across a large study area, dense sampling across a small study area, or sparse sampling across the same small study area. These datasets were a proxy for gene flow (i.e., the response variable) in maximum‐likelihood population effects models that assessed the nature and strength of their relationship with each of five land‐use classes (i.e., potential predictor variables). Comparisons of outcomes were based on the rank order of effect, sign of effect (i.e., gene flow resistance vs. facilitation), spatial scale of effect, and functional relationship with gene flow. The best‐fit model for each dataset had the same sign of effect for hardwood forests, manmade structures, and pine forests, indicating the impacts of these land‐use classes on dispersal and gene flow in *P. mississippi* are robust to sampling scheme. Contrasting sampling densities led to a different inferred functional relationship between agricultural areas and gene flow. Study area size appeared to influence the scale of effect of manmade structures and the sign of effect of pine forests. Our findings provided evidence for an influence of sampling density, study area size, and sampling effort upon inferences. Accordingly, we recommend iterative subsampling of empirical datasets and continued investigation into the sensitivities of landscape genetic analyses using simulations.

## Graphical abstract

We assessed the effects of sampling density and study area size on landscape genetics inferences for a dispersal‐limited amphibian, the Mississippi slimy salamander (Plethodon mississippi), via comparative analysis of nested datasets. Outcomes were interpreted within the context of five possible combinations of congruence among datasets, and our findings provided evidence for an influence of sampling density, study area size, and sampling effort. Given this, we recommend subsampling and reanalysis of empirical datasets and continued investigation into the sensitivities of landscape genetics analyses.

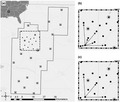

## 1 INTRODUCTION

In the field of landscape genetics, features of the environment that may affect dispersal and gene flow represent potential predictor variables that, once converted to “ecological distances” among local sites, can be compared to corresponding genetic distances among individuals or local populations sampled at those sites (Manel et al., 2003). These comparisons aim to identify abiotic and biotic characteristics of the landscape that influence genetic connectivity, such as riverine or road barriers (Hartmann et al., 2013), agricultural land‐use practices (Costanzi et al., 2018; Goldberg & Waits, 2010; Prunier et al., 2014), or the spatial configuration of preferred or nonpreferred habitat (Vergara et al., 2017). Landscape genetics studies have spanned a broad range of spatial scales (e.g., a study area <40 km^2^ for the Natterjack toad, *Epidalea calamita* [Cox et al., 2017] vs. 250,000 km^2^ for the greater sage‐grouse, *Centrocercus urophasianus* [Row et al., 2015]). In addition to study area size, the spacing between locations where samples are collected (i.e., sampling density) can affect inferences about which ecological variables have the greatest impact on gene flow, as can the scale(s) at which landscape data are summarized (Anderson et al., 2010; Richardson et al., 2016). Thus, study area size and sampling density are important aspects of landscape genetics study design (Anderson et al., 2010; Landguth & Schwartz, 2014; Seaborn et al., 2019). Indeed, questions about sampling design that maximize power for detecting relationships between predictor and response variables apply broadly to field‐based ecological research (Legendre et al., 2002).

Decisions about final sampling density used in landscape genetic studies are often made a priori. While pilot studies have great value (particularly those outside of the study area, so as to avoid pseudo‐replication), they are rarely conducted. As a result, many sampling decisions are instead based on an understanding of the focal species’ dispersal ability, but this may be unavailable to some researchers, and so sampling may simply be conducted opportunistically (Anderson et al., 2010). Typically, species with short dispersal distances are sampled more densely than those for which long‐distance dispersal is common. For example, Peterman et al. (2014) sampled salamanders at locations as close as 75 m apart, whereas Parks et al. (2015) sampled mountain goats 10–25 km apart. Given that fieldwork can be labor‐intensive and expensive, in the case of species with limited dispersal, researchers may be faced with the decision to either conduct their study within a small area so as to avoid exceeding average dispersal distances, or increase the spacing between sampling locations to span steeper gradients of habitat heterogeneity that may be necessary to answer the research questions at hand. For instance, to test the effects of roads on gene flow, ideally, a study area would encompass a moderate to large number of roads of varying sizes (Atzeni et al., 2020; Keller et al., 2014; Richardson et al., 2016)—a condition that may not be satisfied within a small study area. Such decisions about sampling are not trivial, as it is important to design studies that are large enough to detect relevant spatial patterns, yet not so large that these patterns are lost to noise (Landguth et al., 2012; Legendre et al., 2002). Accordingly, there is a need for investigations that explicitly evaluate the impacts of alternative spatial arrangements of sampling sites, using the same focal species, genetic marker set, and landscape setting. This design would enable an examination of the effects of sampling density and study area size on landscape genetics inferences without introducing confounding variables related to intrinsic differences in life‐history traits or mutational processes affecting genetic marker polymorphism, or extrinsic regional differences in abiotic variables and biotic interactions.

Sparse sampling (i.e., where average distances among collection sites far exceed typical movement of individuals) may fail to capture relationships between landscape features and gene flow that are more pronounced for short‐distance dispersal events, such as localized effects of small water bodies. For example, this was seen by Angelone et al. (2011) in a landscape genetics study of the European tree frog (*Hyla arborea*) that assessed the impact of different sampling densities. In their study, the authors separately analyzed pairwise comparisons of breeding ponds that were subdivided into contrasting geographic distance classes. This tiered reanalysis identified different ecological predictor variables as being associated with resistance to gene flow for each distance class examined. The authors considered this outcome to be consistent with the notion that rivers or lakes affected short‐distance dispersal, whereas geographic distance, wetlands, hedgerows, and the density of forests more strongly affected long‐distance dispersal. Another concern about sparse sampling is that it can result in a weaker relationship between genetic distance and the ecological variables that impact gene flow due to stochastic events (e.g., localized extreme weather, interactions with locally invasive or predatory species, and fine‐scale disease spread) that may have accumulated effects between distant sampling sites (Epperson, 2010). For instance, Keller et al. (2013) compared the strength of correlation between genetic and ecologically informed geographic distances among populations of wetland grasshoppers (*Stethophyma grossum*). The strongest fit was found when pairs of sampling locations within close proximity to one another were included in the analyses (i.e., only those up to 3 km apart, the threshold for minimum population connectivity in that study system). Furthermore, the authors found a decrease in model fit when examining widely separated populations, which they suggested may occur because the rarity—and associated stochasticity—of long‐distance dispersal events reduced the ability to detect relationships with ecological variables over such scales. Ultimately, both groups of researchers recommended that sampling locations be spaced no further apart than the average dispersal distance of members of the focal species (also see Anderson et al., 2010).

Just as there may be negative consequences for landscape genetic inferences when sampling is too sparse, this may also be true for very dense, fine‐scale sampling. The logistical trade‐off in which dense sampling is coupled with a smaller study area may create a situation where a representative range of values of one or more ecological predictor variables is not captured by the study design. In such cases, it may become difficult to identify an environmental variable's true impact on gene flow (Keller et al., 2014). This idea is supported by outcomes from a study by Haran et al. (2017), who repeatedly subsampled their dataset of individual‐based genetic distances among pine sawyer beetles (*Monochamus galloprovincialis*) from the Iberian Peninsula. Through a series of partial Mantel tests examining the association between gene flow and environmental variables, they found significant relationships were more likely to be detected when using larger study areas. Briefly, the authors assessed the relationship between environmental variables and genetic distances in over 30,000 alternative demarcations of their study area, ranging from 220 to 1,000 km in diameter. The number of significant relationships between gene flow and high elevation, cooler temperatures, and pine forests was greatest when study area sizes were large (1,000 km diameter), whereas the number of relationships between gene flow and cooler temperatures was highest when study areas were smaller (600 km diameter). While that study highlighted the potential importance of large study areas, its findings are also consistent with Angelone et al. (2011) and Keller et al. (2013) in supporting the idea that the impact of environmental variables on gene flow may be scale‐dependent. Thus, there are reasons for concern regarding overly small study areas, as these may limit researchers’ ability to detect biologically meaningful relationships between ecological predictor variables and gene flow (also see Anderson et al., 2010).

**FIGURE 1:**
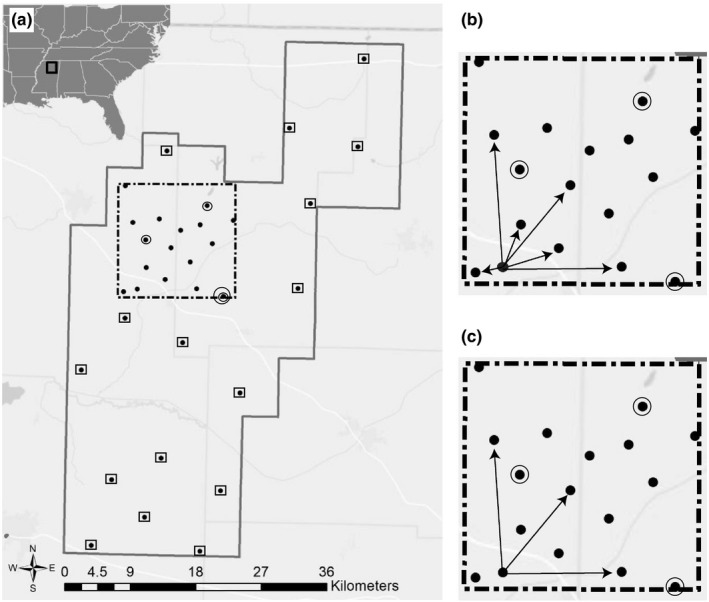
(Preferred choice for graphical table of contents) Sampling locations of *Plethodon mississippi* within Holly Springs National Forest (HSNF), Mississippi, USA. (a) In the sparse sampling across a large study area (630 km^2^), 19 sampling locations (squares with black dots and circles with black dots) were spaced approximately 7 km apart across the entirety of HSNF. Within the small study area (256 km^2^) demarcated by a dashed box, 14 sampling locations (plain black dots) were spaced approximately 3 km apart. The circles with black dots within the small study area indicate locations that were also part of both the large and small study area dataset (inset: map of southeastern United States showing location of HSNF). (b) The dense sampling across a small study area included pairwise genetic distances between individuals from all sampling locations. (c) The sparse sampling across a small study area only included pairwise genetic distances from individuals that were >7 km apart

In the present study, we explored the impact of sampling density and study area size upon landscape genetic inferences for a low‐mobility amphibian, the Mississippi slimy salamander, *Plethodon mississippi* (Highton, 1989). Insights into the effects of sampling design and effort allocation were generated by assessing the relationship between each of five ecological predictor variables (i.e., land‐use types) and gene flow, as measured using individual‐based microsatellite genotypic data, with three sampling schemes: dense sampling across a small study area, sparse sampling across the same small study area (i.e., a reanalyzed subset of the former), and sparse sampling across a large study area that encompassed (and exceeded) the boundaries of the small study area (Figure 1). For each sampling scheme, we created and then ranked by goodness of fit a series of maximum‐likelihood population effects models (MLPE), a type of linear mixed‐effect model that accounts for the nonindependence of pairwise comparisons (Clarke et al., 2002). By comparing the best‐fit models for each of the three sampling schemes, we addressed two questions: (1) Does sparse sampling fail to identify relationships between ecological predictor variables and gene flow that are identified using dense sampling? (2) Are the differences (if any) between inferences obtained from the two contrasting sampling densities a consequence of sampling density alone, or does study area size also play a role?

## 2 METHODS

### 2.1 Focal species and landscape setting

*Plethodon mississippi* is found within bottomland hardwood and wet pine forests of Mississippi, Alabama, and Tennessee, USA, and develops terrestrially without the need to disperse to aquatic environments for reproduction (Petranka, 1998). In northern Mississippi, the species is continuously distributed throughout Holly Springs National Forest (HSNF), a 630 km^2^ federally managed area that contains a mosaic of hardwood forests, manmade structures, agricultural fields, roads, and pine plantations (U.S. Department of Agriculture Forest Service, 2012). To determine the effect of different components of this habitat mosaic on gene flow among populations, it is important that the study area is representative of the forest as whole. However, members of this species are believed to have an average lifetime dispersal distance as short as 100 meters (Wells & Wells, 1976). Given this, sampling that spans such a large area, with locations spaced no further apart than average dispersal distance, would be intractable. Thus, the design of a study focusing on *P. mississippi* in HSNF is an exemplar of the logistical trade‐off between sampling density and study area size that affects many empirical landscape genetic studies of dispersal‐limited herpetofauna.

### 2.2 Study design

To examine the effect of sampling density and study area size upon landscape genetic inferences, nested datasets were created. These contained combinations of two sampling densities and two study area sizes. First, the “Sparse‐Large” dataset consisted of 19 sampling locations (leading to 5,253 individual‐based pairwise comparisons) spaced approximately seven kilometers apart, distributed evenly across an approximately 45 × 70 km region of HSNF (Figure 1a). Second, the “Dense‐Small” dataset consisted of 14 sampling locations (leading to 3,916 individual‐based pairwise comparisons) spaced approximately three kilometers apart, across a 16 × 16 km area nested within the aforementioned large study area, with very similar land‐use composition (Figure 1b). Third, the “Sparse‐Small” dataset was a subset of the Dense‐Small dataset that included individuals from all 14 sampling locations, but only (and all of) those pairwise comparisons between individuals that were greater than seven kilometers apart, thereby matching the distance between sampling locations in the Sparse‐Large dataset (leading to 2,375 individual‐based pairwise comparisons; Figure 1c).

### 2.3 Interpretative framework

Comparisons of similarity among landscape genetic inferences drawn from three nested datasets were used to address our research questions about the relative importance of sampling density and study area size (see Introduction). However, even in the case of very strong similarity, there is little reason to require this outcome to be reflected by *identical* best‐fit models. Accordingly, we focused on using four criteria for which comparisons of the best‐fit models from the three datasets were possible. These were as follows: (1) the rank order of importance of five land‐use classes, (2) the sign of effect upon gene flow, (3) the optimized spatial scale of effect of each landscape variable, and (4) the optimized transformation of each landscape variable. Together, these four criteria enabled objective and meaningful (albeit broad) assessments of similarity among best‐fit landscape genetic models.

Given the three alternative sampling strategies that we considered, for any one of the four criteria of comparison, there are five potential outcomes regarding similarity vs. dissimilarity. First, the effects seen for a given ecological predictor variable may be consistent across all three datasets (Table 1A). This would indicate that outcomes are insensitive to sampling density and study area size. In this case, robust landscape genetic inferences could be obtained from the most logistically tractable study design (i.e., Sparse‐Small), thus providing management‐relevant insights at relatively low expense. A second potential outcome would be a case where sparse sampling in both large and small study areas gave similar inferences, but these strongly differed from those obtained from dense sampling in a small study area (Table 1B). This would suggest that sampling density primarily drives outcomes. Here, the density‐dependent nature of conclusions drawn from a landscape genetic study would mean that the sampling design needs to be tailored to the research question from the outset, based on a priori knowledge. In the absence of such knowledge, an alternative approach would be to sample densely but then reanalyze successively “thinned” datasets to understand the magnitude of impact. A third potential outcome could be where the small study area generated similar inferences irrespective of sampling density, yet these inferences strongly differed from those based on sparse sampling in the large study area (Table 1C). This would suggest that study area size primarily drives outcomes, and as above, the scale‐dependent nature of conclusions would need to be accounted for in the initial sampling design, or explored via reanalysis of successively scaled‐down datasets. The fourth possible outcome would be one in which sparse sampling across the large study area and dense sampling in the small study area produced closely matching inferences, but these contrasted with those obtained from sparse sampling in the small area (Table 1D). We would interpret this to indicate a critical influence of *overall* sample size (i.e., the total number of individual‐based pairwise comparisons), for which there may be threshold effects (i.e., above vs. below *n* pairwise comparisons). If a minimum adequate sampling “effort” exists, this would need to be identified and exceeded, perhaps necessitating a pilot study. The fifth potential outcome would be a scenario where the effects of a given ecological predictor variable strongly differed across all three datasets (Table 1E). This would indicate that a combination of the aforementioned phenomena (i.e., sampling density‐, study area size‐ and/or effort‐dependent outcomes) affected landscape genetic inferences. This latter scenario would suggest that our ability to perform meaningful comparisons across studies is limited, as independent studies will inevitably differ in a least one (or more) aspects of sampling design.

**TABLE 1:** Different combinations of sampling density and study area size, and hypothetical outcomes relating to similarity of landscape genetics inferences among datasets

Interpretation of effect on landscape genetic inferences	Sparse‐Large	Dense‐Small	Sparse‐Small
(A) Insensitive to sampling density and study area size			
(B) Sampling density drives outcomes			
(C) Study area size drives outcomes			
(D) Strong influence of total sample size (± threshold effects)			
(E) Combined effects of density, area size, and/or sample size			

White boxes indicate outcomes are similar to other white boxes, and gray boxes indicate outcomes are dissimilar to both white and gray boxes. A) Similar outcomes are obtained for all three datasets. B) Sparse and dense sampling across a small study area yield similar outcomes, but differ from the sparse sampling across a large study area. C) Sparse samplings across a large and small study area yield similar outcomes, but differ from dense sampling across a small study area. D) Sparse sampling across a large study area and dense sampling across a small study area yield similar outcomes, but differ from sparse sampling across a small study area. E) All models differ. A combination of the effects seen in B) and C) may be the cause of D) or E).

### 2.4 Geographic sampling

To determine where to place the boundaries of the small study area so that it closely represented the land‐use composition of the large (i.e., forest‐wide) study area, we first created a map partitioned into six land‐use classes (i.e., agriculture, hardwood forest, pine forest, manmade structures, water bodies, and wetlands) through a supervised classification in ERDAS Imagine 2014 (Hexagon Geospatial, Norcross, GA, USA) of NASA Landsat 8 satellite imagery (see Supplementary Material for details, and Table S1). The supervised classes were chosen to represent land uses that may impact the dispersal of *P. mississippi*. Bottomland hardwood and wetland areas are the primary habitat for this species (Petranka, 1998). Agricultural areas, manmade structures, and streams have all been associated with resistance to gene flow among populations of some salamander species (Costanzi et al., 2018; Marsh et al., 2007). Finally, putatively suboptimal habitat, such as that provided by pine forests and ridges, has been found to have a relationship with gene flow in a closely related salamander, *P*. *albagula* (Peterman et al., 2014). A 16 × 16 km square polygon shapefile was created in ArcGIS and then moved across the classified raster file until the land‐use class percentages were as close as possible (i.e., within 6.3%) to those found in the large study area (Table S2). As a consequence of optimal placement of the 16 × 16 km square, three sampling locations were shared by the large and small study areas. Accordingly, individuals from these three locations were included in all datasets. To identify differences in the configuration of patches in the small and large study areas, FRAGSTATS v. 4.2 (McGarigal et al., 2012) was used to calculate the patch density, patch cohesion, and correlation length (i.e., a measure that represents the average distance that a randomly placed individual could move without leaving a patch) for each land‐use class (Table S3).

### 2.5 Genetic sampling and analyses

At each sampling location, tail tissue was collected from at least five *P. mississippi* individuals and then stored in 95% ethanol. In total, 183 individuals from 33 locations were sampled (Figure 1). Genomic DNA was extracted using a Qiagen DNeasy Blood and Tissue Kit (Valencia CA, USA), following the manufacturer's recommendations. Eight microsatellite loci reported by Spatola et al. (2013) were used to genotype individuals (Burgess & Garrick, 2020; also see Supplementary Material and Tables S4, S5, and S6 for amplification conditions, allele‐calling approaches, and calculation of genotyping error rates). Given that *P. mississippi* is a continuously distributed species for which there are no apparent discrete local populations within HSNF, for the sole purpose of validating Mendelian inheritance of alleles at each of the microsatellite loci, we grouped individuals into putative populations three different ways. First, all individuals were collectively treated as members of the same population. Second, three sampling locations (two within the large study area only, and one included in both the large and small study areas), from which tissue from 9 to 10 individuals had been collected per location, were each used to represent local populations. Third, the entire dataset was divided by grouping together sampling locations less than 20 km apart (i.e., consistent with the average scale of spatial autocorrelation, see Results), which resulted in eight putative populations. Based on each of these three grouping schemes, we tested for null alleles and departures from Hardy–Weinberg equilibrium, using the package PopGenReport (Adamack & Gruber, 2014) in R (R Core Team, 2019). MICRO‐CHECKER v. 2.2.3 (van Oosterhout et al., 2004) was used to test for departures from linkage equilibrium. Following validation of microsatellite markers, we characterized the full genetic dataset on an individual basis (183 individuals) by calculating percent missing data, number of alleles per locus, and mean allelic richness, using PopGenReport.

Landscape genetic analyses were conducted using an individual‐based genetic distance measure (Shirk & Cushman, 2014). Following Shirk et al. and’s (2017) recommendations for when sample sizes and genetic structure are low, an individual‐based principal components analysis (PCA) was performed using the ade4 package (Dray & Dufour, 2007) in R. Sixty‐four axes of ordination were chosen to achieve a balance between explaining a large amount of variance in the data while also attempting to avoid issues of high dimensionality, where the cost of additional axes outweighs the benefits (Beaumont et al., 2002). The final pairwise genetic distance between all sampled individuals was then calculated using the Euclidean distance between the 64 PCA axes.

To determine the extent of any spatial autocorrelation, we estimated a Mantel correlogram (Oden & Sokal, 1986) for the pairwise genetic distance matrix described above against a straight‐line (Euclidean) distance matrix using the vegan (Oksanen et al., 2019) package in R. The number of distance classes was set at 30 to encompass the smallest distance between two sampling locations. The correlogram was permuted 999 times to generate a null distribution for testing significance, and the Mantel statistic was calculated using a Pearson correlation. Additionally, to determine the geographic extent of any spatial autocorrelation, and determine whether genetic differentiation occurs at multiple spatial scales (Wagner et al., 2005), a semivariogram was created from genetic and Euclidean geographic distances binned into 52 distance classes with a distance interval of 1.5 km using the phylin package (Tarroso et al., 2019) in R. This distance interval was chosen to be smaller than the shortest distance between most sampling locations (i.e., 3 km) while minimizing the number of bins that lacked observations (using this interval, 50 of 52 bins contained observations). If the relationship between geographic and genetic distance differed across distance classes, we would expect this to be evident from different slopes for the nested regressions, and from multiple plateaus within the semivariogram. For the remaining analyses, pairwise genetic distances were calculated separately for the large and small study areas.

### 2.6 Landscape analyses and model testing

The classified land‐use raster (see *Geographic Sampling*¸ above) was used to create a series of rasters for each land‐use class using a moving window analysis in FRAGSTATS v. 4.2 (McGarigal et al., 2012). Moving windows were square, and sizes were designated using the length of one side; thus, a 250 m moving window encompassed 0.0625 km^2^. The value of each pixel in a raster was determined by the percent of the surrounding window that contained a given land‐use class using the PLAND function. Five rasters were created for each land‐use class, with moving window sizes of 100, 250, 500, 750, and 1,000 m. To test for nonlinear relationships between PLAND and gene flow, each of the five rasters per land‐use class was transformed into eight different rasters that encompassed a range of possible positive and negative exponential relationships (see Figure S1) using the ResistanceGA package (Peterman, 2018) in R. Although we acknowledge relationships between gene flow and land‐use variables may have different shapes and maximum resistance levels, for computational tractability, and due to the large size of our study area, we calculated all transformations using maximum resistance set at 100 and shape set at 2.

Pairwise random‐walk distances between individuals within both the large or small study areas were calculated using each raster (i.e., 45 rasters per land‐use class per study area size) using the gDistance package (van Etten, 2017) in R. Random‐walk distances are similar to the resistance distances calculated using the program Circuitscape (McRae, 2006), and when compared to one another, they have been found to differ only in scaling (van Etten, 2017). A raster file with a uniform pixel value of one was also created to calculate random‐walk distance on a homogenous landscape. The latter was used to test the effects of straight‐line geographic distance on genetic distance. To test for correlation between the land‐use classes (i.e., redundancy among potential predictor variables), a series of simple linear regressions were performed between random‐walk distances using the lme4 package in R. If the Pearson correlation coefficient (*r*) between any two land‐use classes was >0.5 (indicating a high level of correlation between the random‐walk distances generated for a pair of land‐use classes), only one of the two land‐use classes was retained. To isolate the effect of each land‐use class on gene flow, the random‐walk distances for each raster were regressed against the homogenous landscape distance using a simple linear regression. The residuals were used in model testing, thereby removing the effect of straight‐line geographic distance.

We analyzed three nested genetic datasets, each associated with a different sampling scheme (see *Study Design*¸ above). For each of these, we separately optimized for the best‐fit spatial scale and transformation of each land‐use class using a series of MLPE models. These are a form of random effects model that are considered robust for individual‐based comparisons (Shirk et al., 2018). Pairwise genetic distances between individuals collected from the same sampling location were removed to prevent skewing models. For example, to optimize the scale and transformation of the agriculture land‐use class, we created 45 separate MLPE models, one for each combination of scale and transformation (e.g., 100 m moving window with a linear functional relationship, 100 m moving window with a monomolecular functional relationship, etc.). For each land‐use class, models were ranked using the corrected Akaike information criterion (AICc; Hurvich & Tsai, 1989). The model with the lowest AICc score for each land‐use class was considered the optimized scale and transformation and used in all subsequent analyses.

Using the optimized scale and transformation for each land‐use class, five univariate models (i.e., each land‐use class alone) and five multivariate models (i.e., combinations of two or more land use classes) were generated to examine how these contributed to variance in genetic distance (Table 2). Each model also included the homogenous landscape distance, as simulations have found that the inclusion of geographic distance in MLPE models increases their model accuracy (Row et al., 2017). Models were ranked using AICc. The best‐fit models were examined to determine the sign of effect for each land‐use class, where a negative sign of effect indicates gene flow facilitation, and a positive sign of effect represents gene flow restriction (Row et al., 2017).

**TABLE 2:** AICc scores for each multivariate maximum‐likelihood population effects model for each set of analyses

Model name	Variables included	Sparse‐Large 103 ind. 5,253 comp. 7 km 630 km^2^	Sparse‐Small 89 ind. 2,375 comp. 7 km 256 km^2^	Dense‐Small 89 ind. 3,916 comp. 3 km 256 km^2^
Full model	GD, A, H, P, M, W	**29,467**	**11,517**	**17,565**
Isolation by distance	GD	30,089	11,693	18,814
Moderate habitat	GD, A, P	29,879	11,769	17,885
Modified habitat	GD, A, M	29,870	11,671	17,868
Forest cover	GD, P, H, W	29,914	11,687	17,935
Agriculture only	GD, A	29,952	11,739	18,046
Manmade structures only	GD, M	29,991	11,708	17,963
Pine only	GD, P	30,025	11,702	17,966
Hardwoods only	GD, H	29,954	11,750	18,015
Wetlands only	GD, W	29,893	11,728	17,847

The number of individuals (ind.), the number of pairwise comparisons (comp.) included in each dataset, the closest distance (km) between sampling locations, and the size of each study area (km^2^) are reported. The lowest AICc scores for each category (i.e., sparse/large vs. dense/small vs. sparse/small) are in bold. Land‐use classes are abbreviated as follows: A = agriculture, H = hardwoods, P = pine, M = manmade structures, and W = wetlands. The effect of geographic distance, calculated using random‐walk distance across a homogenous landscape, is represented by GD.

## 3 RESULTS

### 3.1 Genetic sampling and analyses

**FIGURE 2:**
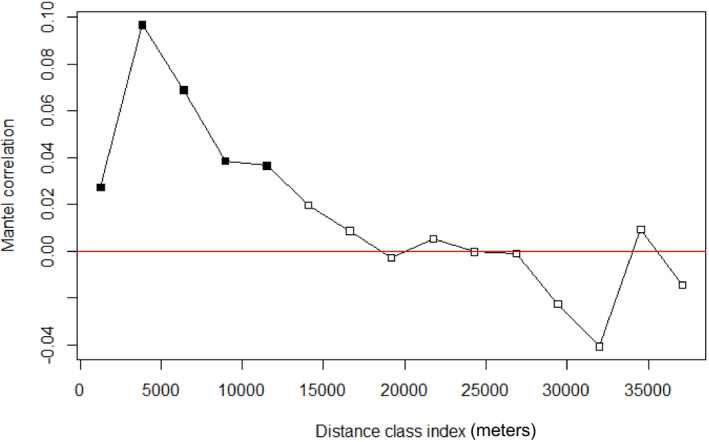
Mantel correlogram of genetic distance permuted against geographic distance. Genetic distance was calculated by first conducting a principal components analysis (PCA) of individual microsatellite genotypes. The final pairwise genetic distance between individuals was then calculated using the Euclidean distance between the 64 PCA axes. Thirty distance classes were tested for 999 permutations to generate significance values. The largest positively correlated and significant distance class, an indicator of genetic neighborhood size, was at 11.5 km (Mantel's *r* = 0.037, *p* = .036)

**FIGURE 3:**
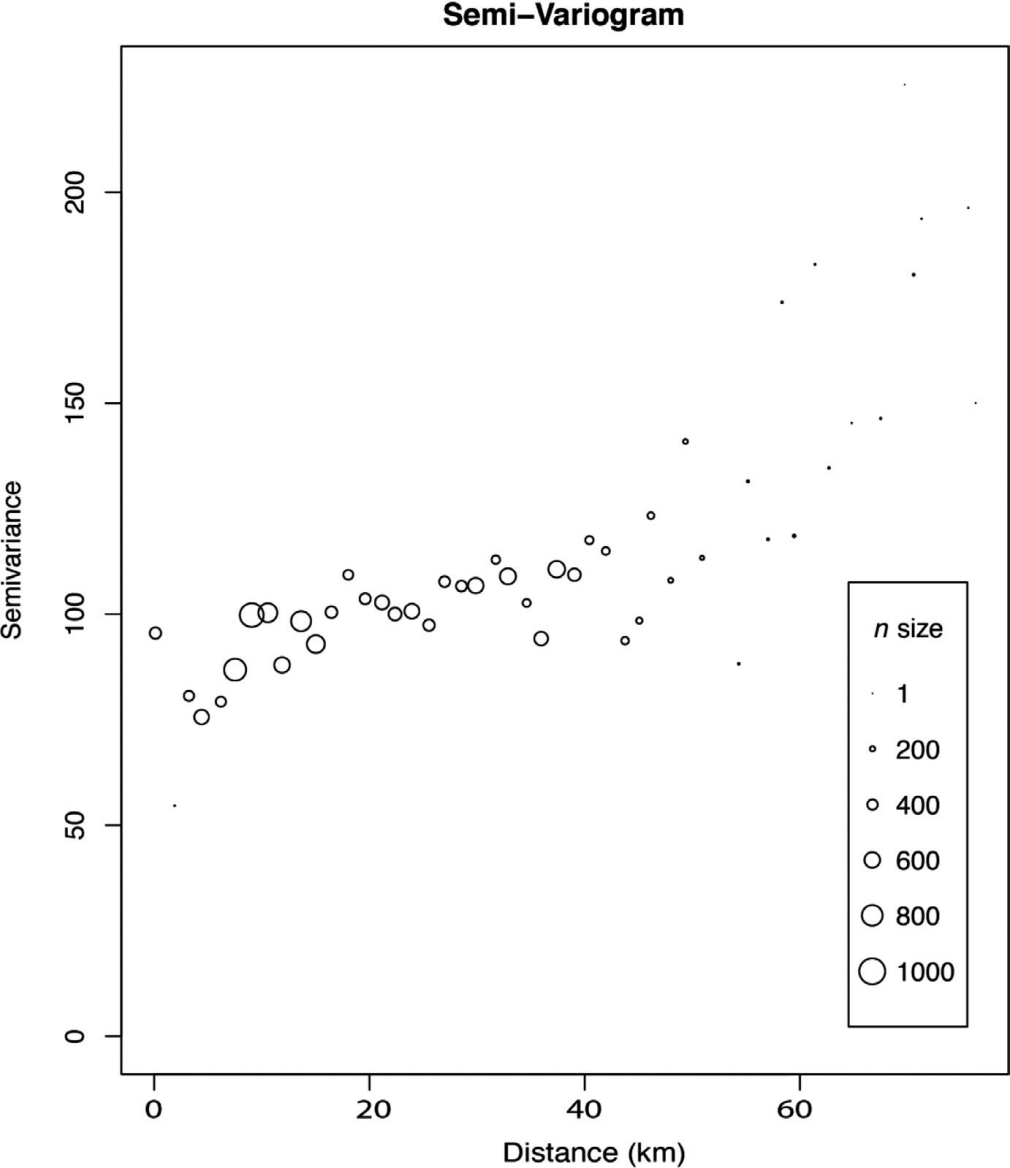
Semivariogram created using pairwise genetic distances and geographic Euclidean distances using 52 distance classes with a distance interval of 1.5 km. The plateau at approximately 10 km indicates that this is the spatial scale over which spatial autocorrelation is the strongest. N size denotes the number of pairwise comparisons within the given distance class

Two microsatellite loci (i.e., PG_QWZ and PG_241) showed evidence of homozygote excess in the three putative populations that were each composed of 9–10 individuals sampled from the same site. However, these loci did not consistently depart from HWE using either of the other two grouping schemes (i.e., when treating all 183 individuals as members of a single population, or when recognizing eight putative populations based on pooling of sites <20 km apart). The same two loci showed some indications of null alleles, but the frequency of nulls was very low (i.e., <0.1). Accordingly, we retained all eight loci in downstream analyses. Based on 183 *P. mississippi* individuals, the number of alleles per locus ranged from 6 to 32, with a mean of 16.5 alleles per locus (Table S6), and the amount of missing genotypic data was 1.8%. The genetic neighborhood size, as shown by the largest distance class at which genetic distance and geographic distance were positively and significantly correlated, was 11.5 km (Figure 2; Mantel's *r* = 0.037, *p* = .036). Consistent with these results, the semivariogram plateaued once, at approximately 10 km (Figure 3).

### 3.2 Landscape analyses and model testing

Tests for nonindependence of potential predictor variables revealed a strong positive correlation (*r* > 0.9) between the wetland and water land‐use classes. Because the primary habitat for *P. mississippi* is wetland areas, the water land‐use class was omitted, and we performed all subsequent analyses using the five remaining classes. For all three nested datasets (i.e., Sparse‐Large, Dense‐Small, and Sparse‐Small), the full model had the lowest AICc and was therefore considered the best‐fit (Table 2).

There were three cases consistent with the interpretation that landscape genetic inferences are insensitive to sampling density and study area size (Table 3A), and all of these related to sign of effect. Specifically, across all three datasets, hardwoods and wetlands were correlated with resistance to gene flow, and manmade structures were correlated with facilitation of gene flow (Tables 3 and 4). The only instance of a sampling density‐dependent outcome (Table 3B) was for the optimized transformation of the effect of agricultural areas, which was the same (i.e., inverse–reverse Ricker) for the Sparse‐Large and Sparse‐Small datasets (Table 4). Our results were consistent with study area size‐dependent outcomes (Table 3C) for two criteria: sign of effect upon gene flow and optimized spatial scale of effect. Pine was correlated with gene flow facilitation in the Sparse‐Large dataset, but the sign of effect for this land‐use class switched to gene flow restriction for the small study area, irrespective of sampling density. Similarly, the optimized spatial scale of effect for manmade structures was 250 m for the Sparse‐Large dataset, compared to 500 m for the other two datasets (Table 4). We observed six instances that suggest an influence of the total number of pairwise comparisons (and the possibility of associated threshold effects), represented by each of the four criteria for comparison (Table 3D). The rank of wetlands, sign of agriculture, scale of agriculture and wetlands, and transformations of manmade structures and wetlands were the same for the Sparse‐Large and Dense‐Small datasets, yet differed from the Sparse‐Small dataset (Table 5). Finally, combined effects of sampling density, study area size, and/or sampling effort were the most common outcome observed across our best‐fit landscape genetic models (Table 3E). For example, for rank order of effect, all land‐use classes except for wetlands showed this pattern. Likewise, for the optimized spatial scale and transformation criteria, hardwoods and pine land‐use classes consistently exhibited unique differences among all nested three datasets (Table 4).

**TABLE 3:** Visual representation of the four criteria: rank, sign (i.e., correlation with gene flow facilitation or resistance), scale, and transformation (labeled “trans.”), for each of the five potential outcomes of congruence detailed in Table 1 at each landscape variable

	Rank	Sign	Scale	Trans.
A. Outcomes insensitive to density and study area size				
Agriculture				
Hardwoods		X		
Manmade Structures		X		
Pine		X		
Wetlands				
B. Sampling density affects outcomes				
Agriculture				X
Hardwoods				
Manmade Structures				
Pine				
Wetlands				
C. Study area size affects outcomes				
Agriculture				
Hardwoods				
Manmade Structures			X	
Pine		X		
Wetlands				
D. Sampling effort affects outcomes				
Agriculture		X	X	
Hardwoods				
Manmade Structures				X
Pine				
Wetlands	X		X	X
E. Density, size, or other factors may influence outcomes				
Agriculture	X	N/A		
Hardwoods	X	N/A	X	
Manmade Structures	X	N/A		
Pine	X	N/A	X	
Wetlands		N/A		

Each “X” indicates the given landscape variable exhibits congruence at the given axis of comparison in the manner described by the hypothetical outcome listed. For example, hardwoods were found to correlate with resistance to gene flow (i.e., the same sign) in all three datasets; thus, the result is consistent with outcome A, where inferences are found to be insensitive to both density and size.

**TABLE 4:** Comparison of the final optimized transformation, scale, and sign of effect for each land‐use class in the Sparse‐Large (dark gray), Dense‐Small (light gray), and Sparse‐Small (white) datasets

Land‐use class	Dataset	Sign	Scale (m)	Transformation
Agriculture	Sparse‐Large	+	500	Inverse–Reverse Ricker
	Dense‐Small	+	500	Inverse Ricker
	Sparse‐Small	‐	1,000	Inverse–Reverse Ricker
Hardwoods	Sparse‐Large	+	500	Inverse Ricker
	Dense‐Small	+	100	Reverse Monomolecular
	Sparse‐Small	+	750	Inverse–Reverse Ricker
Pine	Sparse‐Large	‐	750	Inverse–Reverse Ricker
	Dense‐Small	+	250	Inverse Ricker
	Sparse‐Small	+	1,000	Monomolecular
Manmade structures	Sparse‐Large	‐	250	Inverse Ricker
	Dense‐Small	‐	500	Inverse Ricker
	Sparse‐Small	‐	500	Linear
Wetlands	Sparse‐Large	+	1,000	Inverse Ricker
	Dense‐Small	+	1,000	Inverse Ricker
	Sparse‐Small	+	250	Inverse–Reverse Monomolecular

A positive sign of effect indicates the land‐use class correlates with gene flow restriction, whereas a negative sign of effect indicates gene flow facilitation.

**TABLE 5:** Rank of effect of landscape variables in best‐fit maximum‐likelihood population effects models for each dataset

	Sparse‐Large		Dense‐Small		Sparse‐Small	
Landscape variable rank of effect and model coefficients	W	0.91	W	0.81	P	0.84
	A	0.69	P	0.68	M	−0.74
	H	0.65	M	−0.60	W	0.67
	M	−0.43	A	0.58	H	−0.41
	P	−0.25	H	0.23	A	−0.09

Landscape variables are abbreviated as in Table 4. Model coefficients, or relative contribution of each landscape variable to genetic distance between individuals, are listed next to each landscape variable.

## 4 DISCUSSION

Landscape genetics is a relatively young subdiscipline, and as such, knowledge gaps remain (Richardson et al., 2016). One gap that is common within the field of spatial ecology as well as landscape genetics is an understanding of the impacts that a priori choices regarding sampling density and study area size have upon inferences (Seaborn et al., 2019). Indeed, consideration not only of total sampling effort, but also allocation of that effort in light of the trade‐off between overall spatial extent vs. local density, has been a major focus of recent simulation‐based research (e.g., Landguth et al., 2012; Landguth & Schwartz, 2014). In this study, we used a comparison of landscape genetic models resulting from three alternative sampling strategies for a low‐mobility salamander, *P. mississippi*, to generate insights from datasets that reflect the inherent “noise” embedded in empirical studies. Given that we used ecological predictor variables that were optimized for spatial scale of effect and transformation separately for each dataset, this potentially leads to final best‐fit models with several nonidentical features. As such, there can be many nuanced differences among models, some of which may not directly reflect the impacts of sampling density or study area size. Accordingly, our comparison of models necessarily focused at a relatively coarse level (i.e., primarily the rank ordering of importance of land‐use classes included in each best‐fit model and their overall role in facilitating vs. inhibiting gene flow, and secondarily, optimized scale of effect and transformation). Below, we consider the similarities and differences among inferences drawn from the Sparse‐Large, Dense‐Small, and Sparse‐Small datasets. Within this framework, we highlight the prevalence of five potential outcomes when considering comparisons across all three nested datasets (see *Interpretative Framework* in Methods). We then note limitations of the present work and summarize implications of our findings for sampling design of empirical landscape genetic studies of low‐mobility continuously distributed organisms, such as *P. mississippi*.

### 4.1 Outcomes insensitive to sampling density and study area size

For all three genetic datasets, there was a correlation between the presence of wetlands and resistance to gene flow; the same correlation was also found with hardwood forests. Given that *P. mississippi* individuals often reside in bottomland hardwood forests and wetland areas (Petranka, 1998), it may seem counterintuitive that preferred habitats are associated with resistance to gene flow among populations. However, based on empirical and simulation studies (Keely et al., 2016, 2017), some researchers have reported this same outcome, presumably attributable to individuals choosing to stop or stay in high‐quality habitat areas. In our study, another outcome that was consistent across all three datasets was a correlation between manmade structures and gene flow facilitation. Notably, roads were a significant component of the manmade structure land‐use class in our analyses. Although avoidance of road edges by many amphibians has been documented, Marsh and Beckman (2004) found no effect of forest roads on the presence of *P. glutinosus* (the sister species of *P. mississippi*), supporting the idea that slimy salamanders may move freely across these types of open substrates. This may extend more broadly, given that Prunier et al. (2014) found a correlation between roads and increased gene flow among populations of the alpine newt (*Ichthyosaura alpestris*). As a corollary to counterintuitive idea of slow movement through high‐quality habitat discussed above, an additional interpretation warrants consideration here: individuals may choose to depart or accelerate through low‐quality habitat areas, such that apparent conduits for gene flow may not necessarily be places that also provide protection and/or harbor resident populations.

### 4.2 Sampling density‐dependent outcomes

Based on the design of our study, differences in landscape genetic outcomes that were restricted to comparisons between sparse vs. dense sampling would indicate density dependence of inferences. In some species, dispersal can be divided into two categories: a collection of several small, “routine” movements (i.e., those associated with recurrent processes such local mate‐seeking, or foraging, etc.), or conversely, a singular “special” movement (i.e., directed dispersal away from the natal site; Van Dyck & Baguette, 2005). In a field‐based experimental study that focused on the ringed salamander, *Ambystoma annulatum*, Ousterhout and Semlitsch (2018) identified individuals that traveled only short distances as “residents,” or salamanders that traveled larger distances as “dispersers.” These groups of individuals were found to move differently across the landscape, with dispersers being impacted by habitat type when moving, while residents were not. These contrasting movement behaviors could manifest as different landscape genetic inferences, depending on whether dense vs. sparse sampling was used. However, in the present study, we found only one relatively minor case where this occurred: For the agriculture land‐use class, the Sparse‐Large and Sparse‐Small datasets were both optimized with an inverse–reverse Ricker transformation, whereas the Dense‐Small dataset had an inverse Ricker transformation (Table 4). Interestingly, simulations conducted by Landguth and Schwartz (2014) found that for continuously distributed individuals with limited dispersal, sparse sampling risks overestimating the true magnitude of genetic structure, whereas dense sampling tends to lead to underestimating it. Those authors considered the most likely reasons for this to be either false hierarchical structure in the former case, or inadequate characterization of local variability in the latter. While these issues are certainly noteworthy, given the apparent rarity of sampling density‐dependent outcomes for *P. mississippi*, in our study system, sampling density alone may be less of a concern than initially thought.

### 4.3 Study area size‐dependent outcomes

**FIGURE 4:**
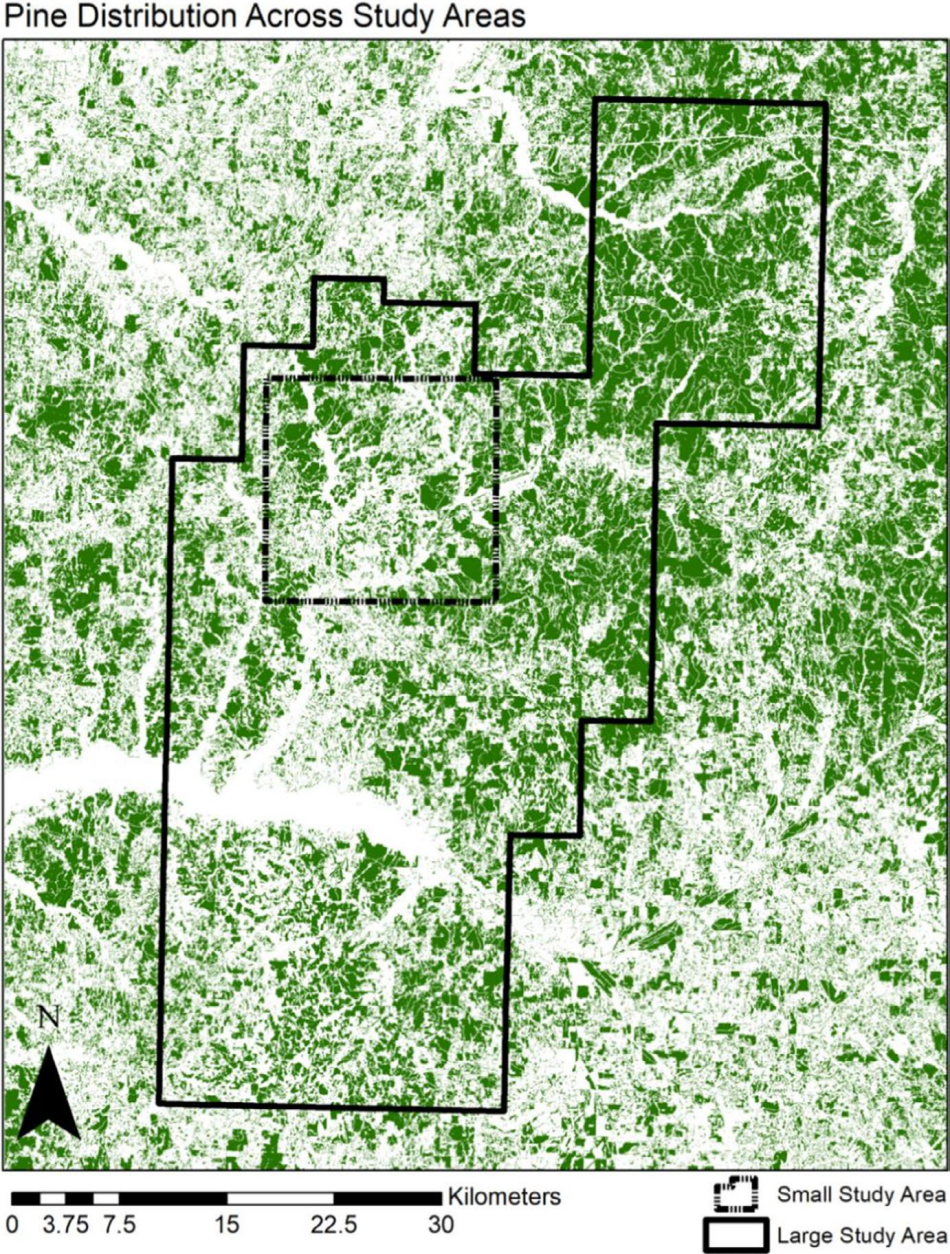
Map illustrating the distribution of the “pine” land‐use class across large and small study areas in Holly Springs National Forest. Burgess and Garrick (2020). The effect of sampling density and study area size on landscape genetics inferences for the Mississippi slimy salamander, *Plethodon mississippi*. *Ecology and Evolution*

Within our study's comparative framework, differences between inferences generated over a large study area (i.e., using the Sparse‐Large dataset) and those from over a small study area (i.e., using the Dense‐Small and Sparse‐Small datasets) suggest that contrasts in study area size may be consequential for landscape genetics studies. One reason for this may simply be that the limited extent of any small study area increases the probability that dispersal and gene flow from outside areas (i.e., unsampled populations that make non‐negligible genetic contributions to the observed data) may obscure or even overwrite signatures of facilitation or resistance by landscape features within the focal area (Anderson et al., 2010; but see Shirk et al., 2021 for a counter‐argument). For *P. mississippi*, we detected two instances of apparent study area size‐dependent outcomes. First, the optimized scale of effect of manmade structures was 250 m for the Sparse‐Large dataset, whereas it was 500 m for the other two datasets. Second, we found a correlation between presence of pine and facilitation of gene flow based on the Sparse‐Large dataset, but the directionality of effect was reversed when instead considering the Dense‐Small or Sparse‐Small datasets (Table 3). It is possible that the configuration and variability of land‐use patches may have contributed to these study area size‐dependent outcomes. Indeed, simulation studies have shown that patch configuration itself is a key component of characterizing heterogeneous landscapes, given that it may not only affect dispersal probabilities, but also time to fixation of alleles, and thus, detection of such events (Cushman et al., 2012; van Strien et al., 2015). In a study of American pika (*Ochotona princeps*), Castillo et al. (2016) found that patch connectivity of a given environmental predictor variable impacted its relationship with gene flow. Other studies have also shown that habitat patch characteristics such as density, cohesion, and correlation length can drive different outcomes in replicated or nested landscape genetic analyses (Burgess & Garrick, 2020; Cushman et al., 2012, 2013; Vergara et al., 2017). When comparing patch configuration metrics, we found that the patch density of pine forests within the small study area was much lower than within the large study area (Figure 4, Table S6). Although patch density can be affected by the size of the area in which it is calculated (McGarigal et al., 2012), in the present study, the difference in pine patch densities was far greater than the differences among the patch densities of agricultural areas, hardwood forests, manmade structures, and wetlands. These findings indicate the effect of pine patches on gene flow among *P. mississippi* populations may be dependent upon the density of those patches across the landscape.

### 4.4 Sampling effort‐dependent outcomes

Strong similarities between one or more components of the best‐fit landscape genetic models that were seen only in the Sparse‐Large and Dense‐Small datasets may be indicative of the effect of overall sampling effort. This could be associated with a threshold effect, where being above vs. below a certain total number of pairwise comparisons drives the result. In our study, such a threshold might lie between 2,375 and 3,916 pairwise comparisons (Table 1). We identified six cases where the pattern of similarity across datasets was consistent with this scenario (Table 5). Of these, the most notable relates to sign of effect: whereas the Sparse‐Large and Dense‐Small datasets showed a correlation between agriculture and gene flow restriction, this relationship switched to gene flow facilitation in the Sparse‐Small dataset (Table 5). However, although the correlation length of agricultural patches in the large and small study areas was similar, composition of agricultural areas differed. Therefore, this is a potentially confounding factor. Indeed, our agricultural land‐use class was composed of an array of crops, including soybeans, cotton, sweet potatoes, and corn. It is possible that the crops grown within the small study area are not representative of those within the large study area, and the relationship between gene flow and agriculture might be dependent upon crop type. That said, given there were five additional examples of sampling effort‐dependent outcomes, we consider the potential for threshold effects of total sample size to be an important consideration. Likewise, simulations by Landguth et al. (2012) showed that the total number of sampled individuals affects the power for detecting impacts of landscape features on dispersal and gene flow for continuously distributed species, although that study modeled an organism with moderate dispersal abilities and used a different inferential framework (i.e., causal modeling) than we did here. Accordingly, we refrain from suggesting that total sampling effort and associated threshold effects are a common phenomenon for low‐mobility species such as salamanders, but it does warrant further investigation.

### 4.5 Combined impacts of sampling density, study area size, and/or sampling effort

Dissimilar outcomes across all three datasets were very common when considering rank ordering of land‐use classes (four cases), and this was also quite common for the optimized scale and transformation included in best‐fit landscape genetic models (two cases each; Table 5). Given that gene flow can only be facilitated or inhibited by a given land‐use type and is therefore a dichotomous feature of our models, the potential for combined impacts of sampling density, study area size, and/or sampling effort could not be assessed for sign of effect. Nonetheless, the prevalence of this dissimilarity across all three datasets suggests that interactions are important, but additional work is needed to dissect the relative contribution of each factor that may be involved. Generally speaking, it is concerning that the rank ordering of land‐use classes (and also optimized scale and transformation) may be difficult to compare across studies owing to the combined impacts of different aspects of study design, as this could limit our ability to draw generalizable conclusions. Indeed, comparisons among unrelated studies are also complicated by inevitable differences in number of loci and their levels of polymorphism (Landguth et al., 2012). However, we suggest that successive reanalysis of subsampled empirical datasets (i.e., those with “thinned” sampling densities, and/or downscaled study area sizes), coupled with metareplication (e.g., Burgess & Garrick, 2020; Castillo et al., 2016; Short Bull et al., 2011), may alleviate some of these issues.

### 4.6 Limitations

In our nested design, we did not include dense sampling across a large study area (i.e., a Dense‐Large dataset) owing the prohibitive size of HSNF. Nonetheless, this would have enabled a more complete exploration of impacts of trade‐offs between different kinds of effort allocation. This is something that can be addressed with simulations (e.g., Landguth & Schwartz, 2014), but we also see value in nested analysis of empirical genetic data that are more realistic in their inherent “noise” (i.e., genotype matrices that may include some or all of the following: alleles from de novo mutations, some genotype scoring error, weak nonindependence among loci, individuals that are not the product of strict outcrossing, and/or departure from 1:1 operational sex ratios). Likewise, we did not include a very fine‐scale sampling strategy in which geographic spacing among sampling locations was equal to or less than the average dispersal distance of individuals. This was because a feasible number of sampling locations would have encompassed a very small area that was unlikely to include all five land‐use classes. However, such fine‐scale sampling has been recommended (e.g., Anderson et al., 2010). Perhaps the most likely impact of this omission from our study is an inability to make inferences on short, near‐contemporary timescales. This is because the inhibiting or facilitating effect of landscape features that arose very recently (or are cyclical) upon direct dispersal of individuals from one location to another requires sampling those spatially proximate locations. Conversely, when sampling is more sparse than average dispersal distances, most of the signal comes from the accumulated multigenerational effects of dispersal and gene flow (i.e., many contributions from unsampled intermediate locations), such that only more permanent or long‐standing landscape features are likely to register (van Strien et al., 2015). Although this may be a missed opportunity in the present study, our exclusive focus on “historical” connectivity (cf. a mixture of contemporary and historical, depending on sampling scheme) may have set up fairer comparisons across different data subsets. Nonetheless, taken together, the aforementioned practical limitations did constrain the number of possible combinations of sampling density, study area size, and sampling effort. We were also limited to only one subset area (cf. multiple replicates), again due to considerations about the logistics of sampling. This is a general restriction of most empirical studies. Indeed, to date, landscape genetic methods have mostly been explored using simulations (e.g., Cushman & Landguth, 2010; Landguth et al., 2012; Landguth & Schwartz, 2014; van Strien et al., 2015), and this includes sampling strategy optimization (Selmoni et al., 2020). While empirical studies can assess interactive and additive forces (Resasco et al., 2017; Seaborn et al., 2019), their use in testing the effect of different methods and/or sampling strategies does have unavoidable limitations. For example, geographic replicates are never identical, and as was the case in the present study, multivariate models created using optimized predictor variables (e.g., land‐use classes) often include predictor variables of different scales and transformations (Castillo et al., 2016; Vergara et al., 2017). We also limited our analyses to assessing the relationship between five land‐use classes and gene flow. There are other environmental variables that may also have relationships with gene flow; however, their addition may potentially increase the number of differences between geographic replicates. While there is certainly scope for improvement, given the importance of optimizing “scale” (see Anderson et al., 2010 for synonyms), the present study serves as a useful case study for identifying real‐world study systems with characteristics that are conducive to a nested sampling design, and for which iterative landscape genetic analyses can be informative about effort allocation and inference sensitivity.

## 5 CONCLUSIONS

The present study revealed an encouraging finding: the directionality (sign) of effect of a given land‐use class on gene flow—whether it acts as a facilitator vs. inhibitor—was commonly found to be insensitive to sampling density and size of the study area. Indeed, this was the case for three of the five land‐use classes considered (i.e., hardwood forests, manmade structures, and wetlands; Table 2). Promisingly, the use of more densely distributed sampling locations (i.e., the Dense‐Small dataset) did not lead to changes in the directionality of the relationship between land‐use classes and gene flow. This suggests that sampling locations for continuously distributed salamander species may be allocated relatively sparsely across large study areas to capture greater environmental variation without failing to identify relationships between environmental variables and gene flow that are seen when using denser sampling. That said, for the three remaining criteria that we used to determine similarity among best‐fit landscape genetic models (i.e., rank ordering of importance, and optimized scale and transformation), we saw evidence for a strong influence upon outcomes of either total sampling effort (coupled with potential threshold effects), or combined effects of sampling density, study area size, and/or sampling effort (Tables 1 and 2). Aside from outcomes of maximum incongruence across nested datasets (Table 3E), we found the greatest number of dissimilarities when considering the effect of overall sampling effort (i.e., total number of pairwise comparisons) on model inferences. Indeed, there may be a need for routine subsampling and reanalysis of empirical datasets (i.e., thinning) to understand the sensitivity of inferences to these aspects of sampling design.

## CONFLICT OF INTEREST

The authors declare that they have no conflict of interest. The findings and conclusions in this article are those of the authors and do not necessarily represent the views of the U.S. Fish and Wildlife Service.

## AUTHOR CONTRIBUTIONS

**Stephanie Burgess:** Conceptualization (lead); Data curation (lead); Formal analysis (lead); Methodology (equal); Writing‐original draft (lead); Writing‐review & editing (equal). **Ryan C Garrick:** Conceptualization (supporting); Data curation (supporting); Formal analysis (supporting); Methodology (equal); Resources (lead); Writing‐original draft (supporting); Writing‐review & editing (equal).

## Supporting information

Supplementary Material

## Data Availability

Genotypic data, R code, and land‐use rasters are available from DRYAD Repository entry found at https://doi.org/10.5061/dryad.h9w0vt4gt
