# Supplementary material for: The effect of sampling density and study area size on landscape genetics inferences for the Mississippi slimy salamander (Plethodon mississippi)
Source: Ecol Evol. 2021 May 1;11(11):6289–304. doi: 10.1002/ece3.7481 (PMC8207395; doi:10.1002/ece3.7481)
Supplement: Supplementary file 1 — Supplementary Material [file ECE3-11-6289-s001.docx]

**Supplementary Material**

*Summary of Imagery Classification*

Spectral bands 3, 4, 5, and 6 were used to conduct a supervised classification of the 30 x 30 m Landsat 8 data into six land use classes (agriculture, hardwoods, manmade structures, pine, weltands, and water) in ERDAS Imagine 2014 (Hexagon Geospatial, Norcross, GA, USA). At least four training polygons were created for each land use class using a combination of high-resolution imagery and selected site visits. Flowline and wetland shapefiles obtained from the US Fish and Wildlife Service National Wetlands Inventory and roads shapefiles derived from the US Forest Service Motor Vehicle Use Map (developed using data from the US Census Bureau) were overlaid onto the classified image using the raster calculator tool in ArcGIS 10.2.2 (ESRI 2011) to classify roads, wetlands, and waterways that may be difficult to identify using aerial classification. Rates of commission and omission errors were calculated for each land use class using 20 randomly selected points and high-resolution imagery and select site visits as ground truth data (Table S1). These error rates were used to calculate a Kappa statistic for the supervised classification, which was 0.85.

Table S1: Assessment of how well the small study area represented land use composition of the large study area, based on rates of commission (land use class is falsely identified) and omission (land use class fails to be identified) errors for each land use class.

| Land Use Class | Commission Rate | Omission Rate |
| --- | --- | --- |
| Agriculture | 10% | 5% |
| Hardwoods | 24% | 5% |
| Pine | 0% | 20% |
| Manmade Structures | 10% | 10% |
| Wetlands | 20% | 20% |
| Water | 6% | 15% |

Table S2: Assessment of how well the small study area represented land use composition of the large study area, based on percent of each land use class found in each.

| Land use class | Large study area (630 km^2^) | Small study area (256 km^2^) |
| --- | --- | --- |
| Agriculture | 8.2% | 9.9% |
| Hardwoods | 31.5% | 25.2% |
| Pine | 41.8% | 45.9% |
| Manmade structures | 4.6% | 4.6% |
| Water | 10.6% | 11.4% |
| Wetlands | 3.3% | 3.0% |

Table S3: Differences in the configuration of patches in the small and large study areas, as measured by patch density (number of patches per 1 km^2^), patch cohesion, and correlation length (a measure of the average distance a randomly placed individual could move before leaving a patch) for each retained land use class.

| Land Use Type | Study Area Size | Patch Density | Patch Cohesion | Correlation Length |
| --- | --- | --- | --- | --- |
| Pine | Large | 17.22 | 95.53 | 562.51 |
|  | Small | 2.83 | 95.21 | 456.59 |
| Agriculture | Large | 8.02 | 92.13 | 295.24 |
|  | Small | 5.03 | 91.14 | 301.82 |
| Hardwoods | Large | 14.04 | 93.24 | 353.68 |
|  | Small | 11.02 | 94.33 | 369.38 |
| Wetlands | Large | 11.59 | 73.56 | 91.30 |
|  | Small | 8.39 | 74.40 | 89.76 |
| Manmade Structures | Large | 1.42 | 98.79 | 5511.04 |
|  | Small | 0.64 | 98.32 | 3513.67 |

*Summary of Laboratory Methods*

We assessed the utility of 27 microsatellite loci developed by Spatola et al. (2013), and found eight loci that reliably amplified for *Plethodon mississippi* from Holly Spring National Forest (HSNF), Mississippi. Polymerase chain reactions (PCRs) were performed in 15 μL volumes, each containing the reagents listed in Table S4. Several samples failed to amplify, and in these cases, amplifications were performed in 15 μL volumes, each containing the reagents listed in Table S5.

PCR products were sent to Yale University’s DNA Analysis Facility on Science Hill for fragment analysis. A ROX-500 size standard was used to estimate allele lengths. We used the software Geneious v.9.1.2 (http://www.geneious.com) to examine the resulting .fsa files and score genotypes. Allele sizes were assigned using bins whose bounds were set -2 base pairs (bp) and +1 bp the size of each allele (all loci contained tetranucleotide repeat motifs). Each .fsa file was viewed and scored individually. MICRO-CHECKER v.2.2.3 (Van Oosterhout et al. 2004) was used to identify alleles sizes that were indicative of stutter peaks or errors created when recording allele sizes (Table S6).

Microsatellite genotyping error rates were calculated by repeating the genotyping of 48 individuals at two loci, thus creating 96 quality control replicates. Five of the repeated genotypes were different, and three of these differences were the result of large allele dropout. This led to a genotyping error rate of 5.2%

Table S4: Reagent list for standard PCRs.

| Reagent | Concentration | Volume (μL) |
| --- | --- | --- |
| 5X Go*Taq* Flexi Buffer (Promega) | - | 3.00 |
| MgCl_2_ (Promega) | 25 mM | 1.20 |
| dNTPs (Promega) | 1.25 mM | 2.40 |
| Bovine Serum Albumin (New England Biolabs) | 10 mg/mL | 0.75 |
| dH_2_O | - | 3.25 |
| Forward primer (with 5' M13 tail) | 1 μM | 0.75 |
| Reverse primer | 10 μM | 0.75 |
| Universal M13 primer (with 5' HEX fluorescent label) | 10 μM | 0.75 |
| Go*Taq* (Promega) | 5U/ μL | 0.15 |
| Genomic DNA | Approx. 40 ng/μL | 2.00 |

Table S5. Reagent list for PCRs of difficult samples

| Reagent | Concentration | Volume (μL) |
| --- | --- | --- |
| Type-It Microsatellite PCR Kit Master Mix (Qiagen) | - | 7.50 |
| dH_2_O | - | 3.75 |
| Forward primer (with 5’ M13 tail) | 1 μM | 0.75 |
| Reverse primer | 10 μM | 0.75 |
| Universal M13 primer (with 5’ HEX fluorescent label) | 10 μM | 0.75 |
| Genomic DNA | Approx. 40ng/ μL | 1.50 |

Table S6. Microsatellite primers for loci developed by Spatola et al. (2013) that were successfully amplified in *Plethodon mississippi*. For each locus, we report forward (F) and reverse (R) primer sequences, annealing temperature (T_a_), number of alleles (N_A_) detected in a sample of 183 individuals and the associated allele size range (in base pairs; bp). The sequence of a 5’ tail used to attach the fluorescently labeled M13 primer is in bold at the 5’ end of each forward primer.

| Locus name | Repeat motif | Primer sequence (5’ to 3’) | T_a_ (°C) | N_A_ | Allele size range (bp) |
| --- | --- | --- | --- | --- | --- |
| PG_43M | AATG | F: **TCCCAGTCACGACGT**AGTCATTGTCAGCTTGCGC  R: GGGAGCTTGCATCAGGAAAG | 55 | 9 | 114-150 |
| PG_POG | AATG | F: **TCCCAGTCACGACGT**ACCTGTATTTCACGCTGCAC  R: CTGCACCTCTCACCCTACTG | 60 | 9 | 238-266 |
| PG_QWZ | AATG | F: **TCCCAGTCACGACGT**TCGTCTGATTATTGCGCTGC  R: ACCTATCTCATCCACCACTGC | 60 | 5 | 163-179 |
| PG_V58 | AATG | F: **TCCCAGTCACGACGT**CTGTGCCACCTTGTTTCCTG  R: TTGTGAGTCTCCTGCCCTTG | 60 | 9 | 146-186 |
| PLAL_241 | AATG | F: **TCCCAGTCACGACGT**CAAAGGTAGGCAATGGTCTCG  R: ACGAGCTAGACCTCTATTTGGG | 55 | 12 | 203-255 |
| PLAL_402 | ATCC | F: **TCCCAGTCACGACGT**AGTGGTGAGGGAGATGGATG  R: TGGACTGTTGCTTTCTTGTGC | 60 | 32 | 111-267 |
| PLAL_545b | AGAT | F: **TCCCAGTCACGACGT**TGGGCCTGGAGCATTACATA  R: GCTTAGTGCAAGGTGTCTTCC | 60 | 28 | 178-394 |
| PLAL_615 | AGAT | F: **TCCCAGTCACGACGT**CCTAAGAGCACGGGACAGAG  R: TATGAGGTCGATCGGTGAGC | 60 | 27 | 154-314 |


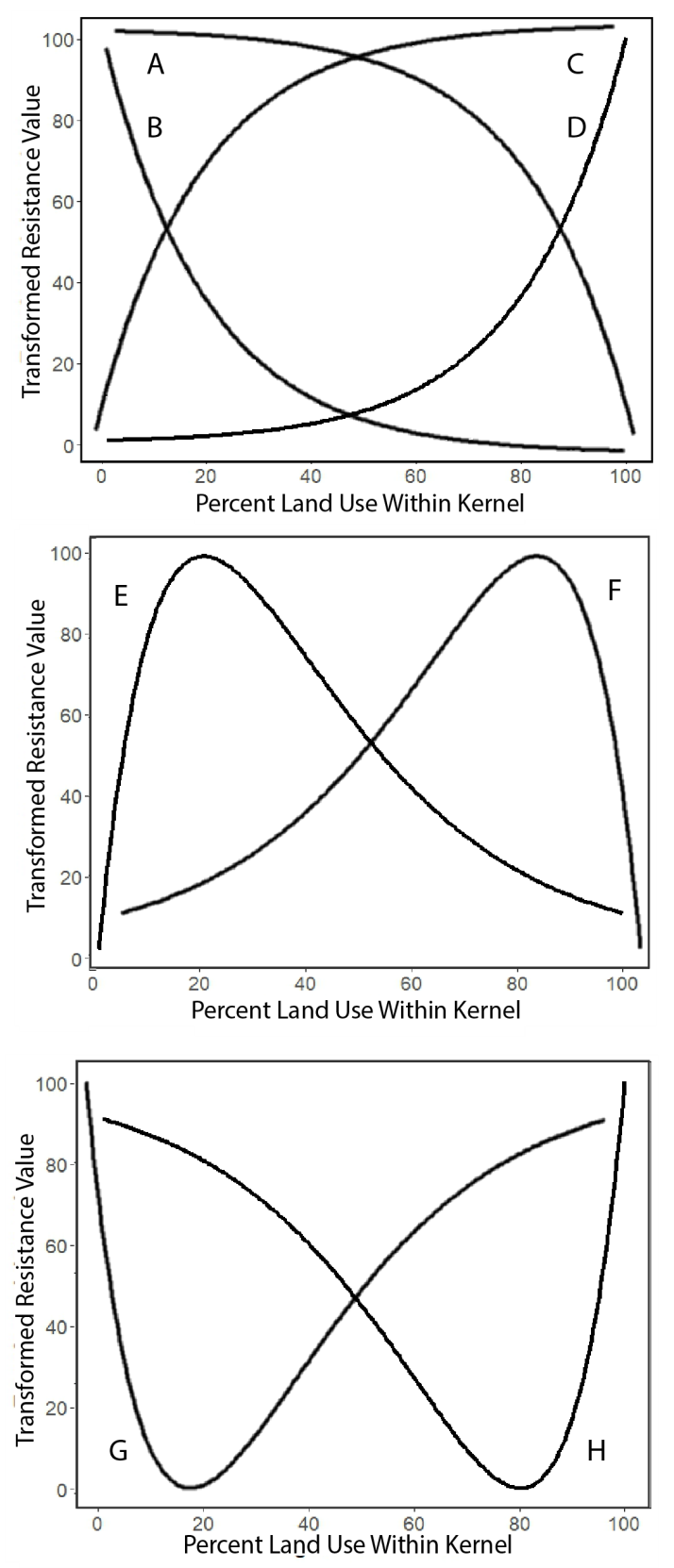


Figure S1: Transformations of original resistance values. The x-axis shows the original resistance value, which indicates the percent of a given land use class within a moving window. The y-axis represents the new resistance value. The alternative transformations are as follows: A) reverse monomolecular, B) inverse monomolecular, C) monomolecular, D) inverse-reverse monomolecular, E) Ricker, F) reverse Ricker, G) inverse Ricker, and H) inverse-reverse Ricker.

**Supplementary References**

Spatola BN, Peterman WE, Stephens NT, Connette GM, Shepard DB, Kozak KH, Selmlitsch RD, and Eggert LS (2013) Development of microsatellite loci for the western slimy salamander (*Plethodon albagula*) using 454 sequencing. Conserv Genet Resour 5:267–270.

Van Oosterhout C, Hutchinson WF, Wills DPM, Shipley P (2004) MICRO-CHECKER: software for identifying and correcting genotyping errors in microsatellite data. Mol Ecol Notes 4:535–538.
